# lncRNA PINK1-AS Aggravates Cerebral Ischemia/Reperfusion Oxidative Stress Injury through Regulating ATF2 by Sponging miR-203

**DOI:** 10.1155/2022/1296816

**Published:** 2022-07-09

**Authors:** Zhong-Bao Yang, Yu Xiang, Mei-Lin Zuo, Li Mao, Guo-Huang Hu, Gui-Lin Song, Md Sayed Ali Sheikh, Li-Ming Tan

**Affiliations:** ^1^The Affiliated Changsha Hospital of Hunan Normal University, Changsha, Hunan 410006, China; ^2^College of Medicine, Hunan Normal University, Changsha, Hunan 410006, China; ^3^Department of Basic Medicine, Changsha Health Vocational College, Changsha, Hunan 410600, China; ^4^Institute of Emergency and Critical Care Medicine of Changsha, The Affiliated Changsha Hospital of Hunan Normal University, Changsha, Hunan 410006, China; ^5^Internal Medicine Department, Cardiology, College of Medicine, Jouf University, Sakaka, Aljouf, 72338¸, Saudi Arabia

## Abstract

Ischemic stroke is a common disease that led to high mortality and high disability. NADPH oxidase 2- (NOX2-) mediated oxidative stress and long noncoding RNA have important roles in cerebral ischemia/reperfusion (CI/R) injury, whereas whether there is interplay between them remains to be clarified. This study was performed to observe the role of lncRNA PINK1-antisense RNA (PINK1-AS) in NOX2 expression regulation. An in vivo rat model (MCAO) and an in vitro cell model (H/R: hypoxia/reoxygenation) were utilized for CI/R oxidative stress injury investigation. The expression levels of lncRNA PINK1-AS, activating transcription factor 2 (ATF2), NOX2, and caspase-3 and the production level of ROS and cell apoptosis were significantly increased in CI/R injury model rats or in H/R-induced SH-SY5Y cells, but miR-203 was significantly downregulated. There was positive correlation between PINK1-AS expression level and ROS production level. PINK1-AS and ATF2 were found to be putative targets of miR-203. Knockdown of lncRNA PINK1-AS or ATF2 or the overexpression of miR-203 significantly reduced oxidative stress injury via inhibition of NOX2. Overexpression of lncRNA PINK1 significantly led to oxidative stress injury in SH-SY5Y cells through downregulating miR-203 and upregulating ATF2 and NOX2. lncRNA PINK1-AS and ATF2 were the targets of miR-203, and the lncRNA PINK1-AS/miR-203/ATF2/NOX2 axis plays pivotal roles in CI/R injury. Therefore, lncRNA PINK1-AS is a possible target for CR/I injury therapy by sponging miR-203.

## 1. Introduction

Ischemic stroke (IS) is a common, aging-related, destructive disease worldwide and is often accompanied by death or disability [1]. The IS incidence is mainly associated with cerebral ischemia and hypoxia caused by cerebral vascular blockage. At present, surgery or thrombolysis is often used in clinical practice to restore the blood flow of the blocked vessels to achieve neuroprotection. However, in this process, oxidative stress injury is often accompanied by reperfusion [2]. Therefore, illustrating the mechanism underlying oxidative stress (OS) injury is very important and urgent for IS prevention and IS therapy, especially illustrating the mechanism of ROS generation under conditions of cerebral ischemia/reperfusion (CI/R) injury.

Our early studies revealed that NADPH oxidase 2 (NOX2) exerts key roles in oxidative stress injury caused by CI/R and is a key enzyme responsible for reactive oxygen species (ROS) [3, 4]. Accumulating evidence proved that NOX2 knockdown or NOX2 enzyme activity inhibition can significantly reduce the production of ROS in brain tissue of experimental stroke (MCAO) model rats, as well as can reduce cerebral infarction and neuronal death [5]. Hence, illustrating the mechanism of NOX2 expression regulation is critical to the comprehension of CI/R oxidative injury, as well as its treatment. ATF2, its full name called activating transcription factor 2, is a transcription factor that participates in gene expression regulation. Previously, ATF2 has been demonstrated involving in the expression regulation of NOX2 via directly affecting the expression level of NOX2 [6]. Therefore, clarifying the role of ATF2 in CI/R injury is crucial to the understanding of NOX2-related oxidative stress.

lncRNA is the RNA whose length > 200 nucleotides, without coding function. Recent evidences demonstrate that lncRNA exerts regulating roles in gene expression and exerts important roles in the pathophysiological process of numerous diseases, including tumors and ischemic stroke [7]. lncRNAs, such as HOTAIR, are reported to have roles in CI/R injury [8, 9]. However, by what mechanism that lncRNA involves in CI/R oxidative injury remains unknown. Evidence from a previous study has revealed that high expression of PINK1-antisense RNA (PINK1-AS) in peripheral blood is related to the occurrence and development of multiple sclerosis [10]. Moreover, Scheele et al. demonstrated that PINK1-AS was involved in the mitochondrial function of nerve cells [10]. In addition, it is reported that PINK1-AS involves in the development of Parkinson's disease [10]. These findings suggest that PINK1-AS may exert important roles in CI/R loss, but its detailed mechanism remains unknown.

MicroRNAs (miRNAs/miRs; 22 nucleotides in length) belong to noncoding RNAs, can suppress its target gene expression, and participate in 70% of gene expression regulation in mammals [11]. Previous studies have shown that lncRNAs, such as maternally expressed 3 and small nucleolar RNA host gene 1, broadly participated in the occurrence and progression of ischemic stroke via competitive bidding with miRNAs [11, 12]. A growing number of evidences demonstrated that lncRNAs, such as lncRNA-H19/miR-148a-3p and lncRNA-OIP-AS/miR-186-5p, are involved in CI/R oxidative stress injury by sponging miRNAs [13].

miR-203 is rich in heart and nervous tissues and is involved in the inflammatory response and cell apoptosis [14]. Previous evidences proved that aberrant expression of miR-203 closely related to cardiovascular diseases; for example, miR-203 insufficiency related to myocardial IR injury or cerebral infarction [15, 16]. In addition, it has been reported that miR-203 could participate in the occurrence and progression of diabetic nephropathy by inhibiting oxidative stress [17]. This suggests that miR-203 exerts important roles in CI/R injury. Using bioinformatics analysis, the present study found that both PINK1-AS and ATF2 may be potential targets of miR-203. Therefore, we hypothesized that the PINK1-AS/ATF2/miR-203 axis may exert important roles in oxidative damage caused by CI/R.

The present study observed the roles of lncRNA PINK1-AS in CI/R oxidative injury and its possible underlying molecular mechanism. As far as we know, this study first revealed that PINK1-AS aggravated CI/R injury via regulation of the expression of the ATF2/miR-203 axis.

## 2. Materials and Methods

### 2.1. Animal Experiments

Totally, 16 SD rats were used for this study. These SD rats all are male, with a weight of 250 g and with an age of 8 weeks. The animals were fed in the following condition: 25°C ambient temperature, 65% humidity, and days alternates with night. The MCAO rat model was constructed following the methods by Yang et al. [18]. Briefly, lightly anesthetize by injecting pentobarbital sodium (3 percentage) intraperitoneally, ischemia for 2 h by occluding the left internal carotid artery using a nylon filament, and reperfusion for 20 h by removing the filament and restoring the blood. Following that, the brain tissue was sampled for related analysis, such as TTC staining and gene expression. The animal experiments adhere to the rules put forward by NIH [19], and this study was approved by the Ethics Committee of Hunan Normal University.

### 2.2. Cell Culture

For in vitro experiments, a neuron cell line (SH-SY5Y cell) was used. The cells were held in conditions of 37°C, 5% CO_2_, and 95% air. DMEM replenished with 10% FBS (Thermo Fisher) and penicillin/streptomycin (100 U/mL) were used for cell culture. A total of 5 × 10^5^ cells were seed into 12-well plates for functional study.

### 2.3. Hypoxia/Reoxygenation (H/R) Cell Model

An in vitro ischemic stroke model was established to mimic the CI/R injury. The SH-SY5Y cell was used for the H/R inducement. For hypoxia, the cells were incubated with DPBS, a balance salt solution without glucose, and placed in a hypoxic environment (37°C, 0% O_2_, and 5% CO_2_) for 5 h. For reoxygenation, the DPBS was removed first; then, the cells were incubated with DMEM culture in normal conditions for 20 h.

### 2.4. Bioinformatics Analysis Prediction

Two online bioinformatics software were used for interaction prediction between targets; they are TargetScan and miRBase.

### 2.5. miR-203 Mimic Transfection

The miR-203 mimics (5′-GAUCACCAGGAUUUGUAAAGUG-3′) and negative controls (NC) were used for function assays of miR-203 in neurons. Lipofectamine® 2000 (Thermo Fisher) was used for transfection reagent. The concentration of miR-203 mimics used for transfection experiment is 100 nm/L.

### 2.6. Gene Knockdown

Small interfering RNAs (siRNAs) against lncRNA PINK1-AS, ATF2, and the siRNA NCs were used for function assay of lncRNA PINK1-AS and ATF2 in neurons. For transfection solution preparation, the Lipofectamine 2000 and siRNAs against lncRNA PINK1-AS (R: AAGAGAUUUCAUGUCUAACUA; R: GUUAGACAUGAAAUCUCUUCC) or ATF2 (F: ACAAUGACACUGUCAUUACGU; R: GUAAUGACAGUGUCAUUGUGG) or siRNA NC were mixed up. For transfection, the cells were incubated with transfect mixture containing 100 nm siRNA against lncRNA PINK1-AS or ATF2 for 24 h. Following that, cells were sampled for related assays, such as mRNA and protein expression.

### 2.7. Luciferase Reporter Assay

To investigate the interplay between PINK1-AS and miR-203 or between ATF2 and miR-203, a luciferase reporter experiment was conducted. The pCMV-CRB-EGFP (Promega Corporation) was used as for vector plasmid and the 3′UTR sequence of a certain gene (PINK1-AS or ATF2) containing the seed sequence was cloned into it. The recombinant plasmid of PINK1-AS was designated as lnc-PINK1 wild type (WT) or lnc-PINK1 mutant (MU), depending on the seed sequence which is wild type “GUAAAGU” or mutant “GUACCGU.” Similarly, the recombinant plasmid of ATF2 was designated as ATF2-WT (wild-type seed sequence: CAUUUCA) or ATF2-MU (mutant seed sequence: CAUGGCA). The SH-SY5Y cells were incubated with these recombinant plasmids and miR-203 mimics for 24 h of transfection. Following that, the relative luciferase expression was measured.

### 2.8. p-PINK1-AS Plasmid

lncRNA PINK1-AS sequence segment containing the putative binding site for miR-203 (between the sites 1961 and 2170), 210 nucleotides in length, was inserted into the restrictive sites Sac I of the pCMV-CRB-EGFP vector plasmid to construct a recombinant plasmid which was designated as p-PINK1-AS and validated by electrophoresing (figure S1). Then, the p-PINK1-AS were used for overexpression analysis of a gene.

### 2.9. Apocynin Treatment

To observe the effect of inhibition of NOX enzyme on oxidative stress, SH-SY5Y cells were treated with apocynin (sigma), a NOX inhibitor, for 24 h. The concentration of apocynin used in this study is 10^−4^ mol/L. Following that, the cells were sampled for relevant analysis, such as gene expression measurement and other assays.

### 2.10. Determination of NOX Activity

The total protein was extracted from the samples, and it was utilized for NOX activity measurement. A NADPH oxidase assay kit based on colorimetry (Genmed, Shanghai) was used for total NOX enzyme activity determination. The reaction solution that consists of protein extraction, oxidized cytochrome *c* (2 *μ*L), and NADPH (2 *μ*L) was prepared. Following that, the spectrophotometer was utilized to measure the AD value of the solution at 550 nm and the NOX activity was calculated.

### 2.11. Measurement of Caspase-3 Activity

The total extracted protein from the samples was utilized for NOX activity measurement. For caspase-3 activity measurement, a caspase-3 activity assay kit (Beyotime) was utilized. Briefly, make a reaction solution that comprises protein extractions, caspase-3 substrate, and Ac-DEVD-pNA. The 100 *μ*L reaction system was established as follows: 40 *μ*L of detection buffer, 50 *μ*L of protein to be tested, and 10 *μ*L of substrate. Then, the reaction solution was placed into the place where the temperature is 37°C for a 60 min reaction in dark. Following that, the absorbance value at 405 nm was detected using a reader. The caspase-3 enzymatic activity was calculated according to kit instruction.

### 2.12. ROS Measurement

For measuring ROS content in brain tissues or cells, a ROS assay kit based on DCFH-DA (Beyotime) was used. Following the kit instructions, first, prepare the brain tissue homogenate or the cells; then, these tissue or cell preparations were mixed with DCFH-DA (10 *μ*M) and mixed up fully by vortex. After that, the reaction solution was placed into the place where the temperature is 25°C and in the dark. After a 20 min reaction, the fluorescence signal intensity was measured.

### 2.13. TUNEL/Hoechst Double Labeling

For brain tissue apoptosis evaluation, a double-labeling staining was conducted, that is, TUNEL staining in conjunction with Hoechst staining. Briefly, the brain sections have undergone the following processes: (1) formaldehyde fixing: brain slices with 5 *μ*m thick was immersed with 4% *w*/*v* formaldehyde firstly; then, the brain slices were placed into the place where the temperature is 25°C and in the dark for full reaction; the total reaction time is 10 min; (2) washing: remove the fixing solution (4% *w*/*v* formaldehyde) and add into PBS to remove residual fixative fluid. To make sure the fixative fluid was get rid of thoroughly, the slices were washed twice or three times with PBS; (3) postfixing: then, the brain slices were immersed with formaldehyde and acetic acid and placed in the dark at 4°C for 5 min; (4) washing: remove the postfixing solution (formaldehyde and acetic acid) firstly, then adding into PBS to remove residual fixative fluid. To make sure the fixative fluid was get rid of thoroughly, the slices were washed twice or three times with PBS; (5) TUNEL reaction: then, the slices were incubated with 50 *μ*L of TUNEL detection liquid which consists of deoxynucleotide transferase (TdT enzyme) and fluorescent labeling solution and placed in the dark at 37°C for 60 min; (6) washing: remove the deoxynucleotide transferase solution firstly and then, rinse twice with PBS to remove residual solutions; (7) Hoechst nuclear staining: then, the slices were incubated with Hoechst 33342 staining and placed into the place where the temperature is 25°C and in the dark for full reaction, the total reaction time is 5 min; (8) washing: remove the Hoechst 33342 staining firstly, and rinse twice with PBS to remove residual solutions; (9) seal slice: adding antifluorescence quenching sealing liquid on slices to prevent fluorescence quenching. At the end, images were taken by a epifluorescence microscope (Nikon) and the TUNEL-positive cells were calculated.

### 2.14. Hoechst Staining

For cell apoptosis analysis, the Hoechst staining was conducted. Briefly, the SH-SY5Y cells have undergone the following handles: (1) fixing: cells were incubated with 4% paraformaldehyde first, and it was placed into the place where the temperature is 25°C and in the dark for full reaction; the total reaction time is 10 min; (2) washing: remove the fixing solution (4% formaldehyde) firstly, and then, rinse twice with PBS to remove residual fixative fluid thoroughly; (3) Hoechst staining: then, the cells were immersed into Hoechst 33258 staining solution (Beyotime) and it was placed into the place where the temperature is 25°C and in the dark for full reaction; the total reaction time is 5 min; (4) washing: remove the Hoechst 33342 staining firstly, and rinse twice with PBS to remove residual solutions; (5) adding antifluorescence quenching liquid to prevent fluorescence quenching. Then, the images were taken using a fluorescence microscope. Following that, the number of apoptotic cells was calculated.

### 2.15. Real-Time PCR

To observe the gene expression, real-time PCR was used in this study. Fist, samples were mixed with the TRIzol® kit (Takara Biotechnology Co., Ltd.) and blended by vortex. Following the kit instruction, the total RNA extraction process is as follows: (1) cell collection: the brain tissue homogenate and the cells were transferred to a 1.5 mL tube and suspended with PBS; then, the suspension was centrifuged to get the cell pellet (3000 r/min, 10 min); (2) RNA extraction: the cell pellets were mixed with TRIzol® reagent and blended by vortex; then, it was placed into the place where the temperature is 4°C and in the dark for full reaction; the total reaction time is 30 min; (3) centrifuge: the RNA extraction solution was centrifuged at 12000 r/min for 15 min; (4) rinsing: remove the supernatant, and collect the RNA pellet first; then, rinse with ethanol twice to remove residual extraction thoroughly; (5) RNA dissolution: then, the RNA pellet was dissolved into RNA enzyme-free water for subsequent assay. A NanoDrop One spectrophotometer was used for RNA purity and RNA concentration determination. The RT kit (Takara) was used for getting the cDNA. According to the kit instruction, a 10 *μ*L reverse transcription reaction system was established as follows: (1) for miRNA: 2 *μ*L of primer mixture, 2 *μ*L of 5×PrimeScript buffer, 0.5 *μ*L of reverse transcription enzyme, 1.5 *μ*L of dH_2_O without RNase, and 400 ng of total RNA; (2) for mRNA: 0.5 *μ*L of random, 0.5 *μ*L of Oligo dT, 2 *μ*L of 5 × PrimeScript buffer, 0.5 *μ*L of reverse transcription enzyme, 2.5 *μ*L of dH_2_O without RNase, and 400 ng of total RNA. ABI 7300 plus system and the SYBR™ Green PCR kit (Takara) were utilized for real-time PCR reaction. Following the kit instruction, a 10 *μ*L real-time PCR reaction system was established as follows: (1) for miRNA: 5 *μ*L of SYBR Premix Ex Taq™, 0.4 *μ*L of primer mixture, 0.2 *μ*L of ROX Reference Dye (50×), 2 *μ*L of cDNA, and 2.4 *μ*L of RNase Free dH_2_O. Real-time PCR reaction parameters were set as follows: 95°C, 30 s; 95°C, 5 s; and 60°C, 31 s, 40 cycles. The 2^-*ΔΔ*Cq^ method was utilized to express gene expression analysis [20]. *β*-Actin and U6 serve as internal reference. The primers for real-time PCR are shown in Table 1.

### 2.16. Western Blotting

To observe the gene expression, western blotting assay was used in this study. Total protein was extracted using a commercial kit (Beyotime). Following the manufacture's instruction, protein extraction mainly includes the following process: (1) cell collection: the brain tissue homogenate and the cells were transferred to a 1.5 mL tube and suspended with PBS; then, the suspension was centrifuged at 3000 r/min for 15 min; (2) cell lysis: remove the supernatant, and get the cell pellet first; then, the cell pellet was mixed with western and IP lysis containing 0.1% PMSF (a proteinase inhibitor) and mixed up fully by vortex; then, it was placed into the place where there is ice and in the dark for full reaction; the reaction time is 40 min; (3) centrifuge: the cell lysis was centrifuged at 12000 r/min for 15 min; (4) protein collection: collecting the supernatant for subsequent assay. The protein concentration was measured utilizing a commercial kit (Beyotime). After that, the protein was denatured at 99°C. For western blotting assay, protein from each sample has undergone the following handles: (1) protein separating: a 10% SDS-PAGE gel was prepared first; then, 30 *μ*g was added into the lane of the10% SDS-PAGE gel; after that, the gel was placed on a electrophoresis apparatus to separate the target protein; (2) blotting: the gel containing the target protein was rinsed with transfer buffer TBST and placed on a transfer membrane device; then, the target protein was blotted to PVDF membranes; (3) occlusion: preparation of 5% fat-free milk using TBST first; then, the PVDF membranes were incubated with this milk at 25°C for occlusion; (4) washing: the membranes were rinsed with PBS twice to remove the residual milk, each for 5 min; (5) incubating with primary antibodies: the PVDF membranes were immersed into diluted primary antibody solution first; then, it was placed into the place where the temperature is 4°C and in the dark for full reaction; the reaction time is 16 h; (6) washing: the membranes were rinsed with water twice to remove the residual antibodies, each for 5 min; (7) incubating with secondary antibodies: the membranes were immersed into diluted secondary antibody (Beyotime) solution; then, it was placed into the place where the temperature is 25°C for full reaction; the reaction time is 2 h; . (8) washing: the membranes were rinsed with water twice to remove the residual antibodies, each for 5 min; (9) ECL solutions and a chemical imaging system (Bio-Rad) were used for band signal detection. ImageJ 1.43 software was utilized for densitometric measurement. The primary antibodies against rabbit anti-ATF2 (sc-242) anti-caspase-3 (sc-7272), anti-NOX2 (sc-130543), or *β*-actin sc-47778) (all Santa Cruz Biotechnology, Inc.) were diluted by 1 : 1,000. The results were normalized with *β*-actin.

### 2.17. Statistical Analysis

GraphPad Prism software (version 7) was used for statistical analysis. The discrepancy between groups was assessed using *t*-test or one-way ANOVA. The correlation analysis was analyzed using a Pearson correlation coefficient. The data were expressed as mean ± SD, and all experiments were repeated 3 times. *P* < 0.05 means there is a statistical difference.

## 3. Results

### 3.1. Increased lncRNA PINK1-AS Expression Is Associated with CI/R Injury in Rats

**Figure 1:**
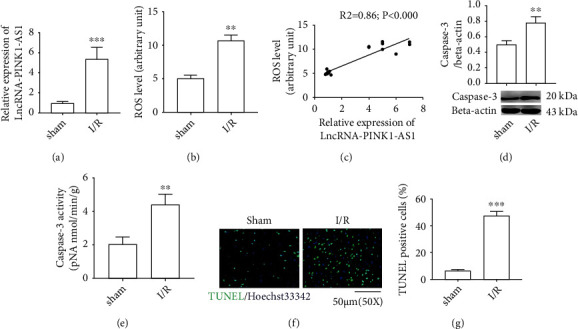
The increased lncRNA PINK1-AS associates with CI/R oxidative stress injury in rats. (a) mRNA level of lncRNA PINK-AS1; (b) ROS level; (c) correlation analysis; (d) protein expression of caspase3; (e) caspase3 activity; (f) representative images of TUNEL/Hoechst double staining; (g) the number of TUNEL-positive cell. The correlation between groups was analyzed by Pearson's correlation coefficient. ^∗∗^*P* < 0.01 vs. sham; ^∗∗∗^*P* < 0.001 vs. sham.

For observing the role of lncRNA in CI/R injury, we firstly determined the differentially expressed lncRNA in brain tissues. As the results have shown, compared with the control, the lncRNA PINK1-AS expression level was obviously increased in the CI/R injury group (Figures 1(a) and 1(b)). Next, the ROS levels were detected and it was found that these were obviously increased in the CI/R group (Figure 1(b)). Subsequently, the correlation between lncRNA PINK1-AS expression and the generation of ROS was analyzed, and it was found that there was a positive correlation between lncRNA PINK1-AS expression level and ROS level (Figure 1(c)).

The next experiments were conducted for cellular apoptosis analysis in brain tissues. As the present result has shown, the expression level of caspase-3 and its enzyme activity in brain tissues in the CI/R group were significantly increased versus those in control (Figures 1(d) and 1(e)). Consistently, the TUNEL assay has shown that TUNEL-positive cell number was obviously augmented in the CI/R group. This indicated that the cells in the CI/R brain tissues underwent apoptosis (Figures 1(f) and 1(g)). These data suggested that the upregulation of PINK1-AS during CI/R may be closely associated with oxidative stress injury in brain tissues.

### 3.2. lncRNA PINK1-AS Knockdown Reduces Oxidative Stress in H/R-Induced SH-SY5Y Cells

**Figure 2:**
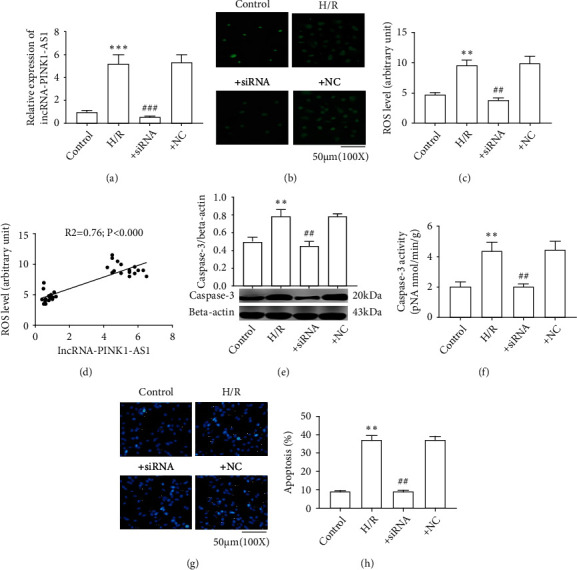
PINK1-AS knockdown reduced oxidative stress in H/R-treated SH-Y5Y cells. (a) mRNA level of lncRNA PINK-AS1; (b) presentative images of ROS; (c) ROS level; (d) correlation analysis; (e) protein expression of caspase3; (f) caspase3 activity; (g) the detection of cell apoptosis by Hoechst staining. (h) percentage of cell apoptosis. The correlation between groups was analyzed by Pearson's correlation coefficient. ^∗∗^*P* < 0.01 vs. control; ^∗∗∗^*P* < 0.001 vs. control; ^##^*P* < 0.01 vs. H/R; ^###^*P* < 0.001 vs. H/R.

To further observe the relationship between PINK1-AS and CI/R oxidative stress injury, an *in vitro* cell model was constructed. In accordance with the animal experiment results, H/R induction obviously upregulated the expression level of PINK1-AS and increased the production of ROS in SH-SY5Y cells (Figures 2(a) and 2(b)). Consistently, it was also found that there was a positive correlation between PINK1-AS expression level and ROS levels in SH-SY5Y cells (Figure 2(c)).

The subsequent investigations were conducted to observe the effect of PINK1-AS knockdown on oxidative stress in SH-SY5Y cells. As the present data has shown, the siRNA against PINK1-AS significantly reduced the expression level of PINK1-AS and the ROS levels in H/R-treated cells, while siRNA NC had no effect on it (Figures 2(a) and 2(b) and Figure S2). Next, the cell apoptosis was detected in SH-SY5Y cells. The evidence demonstrated that H/R induction obviously increased the expression level of caspase-3, as well as its enzyme activity in SH-SY5Y cells, while PINK1-AS knockdown significantly reversed these effects (Figures 2(d) and 2(e)). Furthermore, the Hoechst staining results revealed that H/R visibly induced cell death of SH-SY5Y cells, while PINK1-AS knockdown significantly inhibited cell apoptosis caused by H/R (Figures 2(f) and 2(g)). These results suggested that PINK1-AS has important roles in CI/R injury.

### 3.3. lncRNA PINK1-AS and ATF2 Are Targets of miR-203

**Figure 3:**
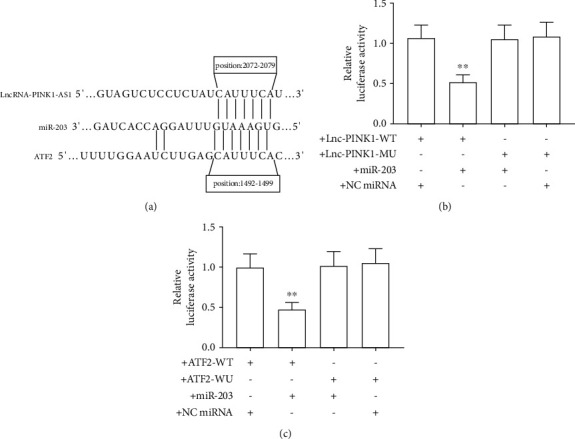
lncRNA PINK1-AS and ATF2 are targets of miR-203. (a) sketch map of bioinformatic prediction; (b, c) relative luciferase activity. ^∗∗^*P* < 0.01 vs. +lnc-PINK1-WT and +NC miRNA.

Considering that the common biological function of lncRNAs is to act as competing endogenous RNAs for miRNAs with coding genes, the current study then performed bioinformatics analysis to predict the possible targets of PINK1-AS. As shown in Figure 3(a), there was an interaction between PINK1-AS, ATF2, and miR-203. We then carried out reporter experiments to conform this hypothesis. As the present data has shown, miR-203 mimic obviously deduced the luciferase expression in SH-SY5 cells that expressed lnc-PINK1-WT or ATF2-WT when compared with the control, but without obvious effect on the luciferase expression in SH-SY5Y cells that expressed lnc-PINK1-MU or ATF2-MU. Different from the result of miR-203, the negative miRNA has no effect on the relative luciferase activity in SH-SY5Y cells that were transfected with a wild plasmid (lnc-PINK1-WT or ATF2-WT) or that were transfected with a mutant plasmid (lnc-PINK1-MU or ATF2-MU). These data fully suggested that PINK1-AS and ATF2 were direct targets of miR-203 (Figures 3(a) and 3(b)).

### 3.4. miR-203 Inhibits Oxidative Stress Injury by Targeting ATF2

**Figure 4:**
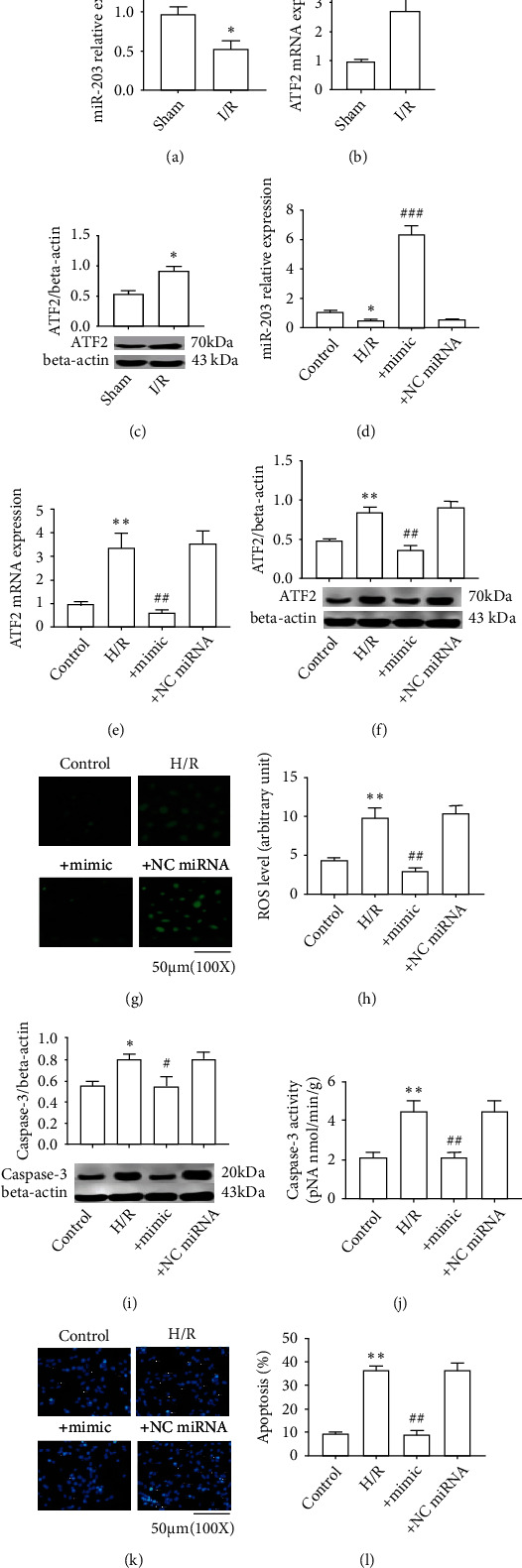
miR-203 inhibits oxidative stress injury by targeting ATF2. (a) miR-203 expression (rat); (b) ATF2 mRNA expression (rat); (c) ATF2 protein expression (rat); (d) miR-203 expression (SH-SY5Y cell); (e) ATF2 mRNA (SH-SY5Y cell); (f) ATF2 protein expression (SH-SY5Y cell); (g) presentative images of ROS; (h) ROS level; (i) the expression level of caspase3 protein; (j) caspase3 activity; (k) the detection of cell apoptosis by Hoechst staining. (l) percentage of cell apoptosis. The correlation between groups was analyzed by Pearson's correlation coefficient. ^∗^*P* < 0.01 vs. sham or control. ^∗∗^*P* < 0.01 vs. control or sham; ^#^*P* < 0.05 vs. H/R; ^##^*P* < 0.01 vs. H/R; ^###^*P* < 0.001 vs. H/R.

Considering that ATF2 is an important transcription factor for NOX2, we speculated that there has a correlation between NOX2 and ATF2 or gene that may be regulating ATF2. Our next investigations analyzed related gene (ATF2 and its target miR-203) expression in brain tissues or SH-SY5Y cells. The present data has shown that miR-203 was significantly downregulated in CI/R model rats when compared with the sham (Figure 4(a)). In contrast, ATF2 was found obviously upregulated in the CI/R group versus the sham (Figures 4(b) and 4(c)). Consistently, the present data has shown that miR-203 was obviously downregulated in H/R-induced SH-SY5Y cells versus control (Figure 4(d)). It is different from miR-203; ATF2 was obviously upregulated in H/R-induced SH-SY5Y cells (Figures 4(e) and 4(f)).

Our next experiments carried out to observe whether miR-203 can affect the oxidative stress injury in SH-SY5Y cells. As the present data has shown, miR-203 mimics obviously upregulated miR-203 expression in H/R-induced SH-SY5Y cells, while miRNA NC has no obvious effect on miR-203 expression (Figure 4(d) and FigureS2). This indicated that the miR-203 mimics functioned correctly and subsequent experiments could be performed. As shown in Figures 4(e) and 4(f), the miR-203 mimics significantly downregulated ATF2 expression (mRNA and protein) in H/R-induced SH-SY5Y cells, whereas the negative miRNA has no obvious effect on miR-203 expression in H/R-induced cells. Next, the ROS levels were measured and it was found that miR-203 mimics could obviously reduce the ROS levels in H/R-induced cells, while the negative miRNAs have no obvious effect on the ROS level in H/R-included cells (Figures 4(g) and 4(h)). In accord with the result of ROS levels, the expression of caspase-3 and its enzyme activity in H/R-induced SH-SY5Y cells were found obviously increased when compared to those in the control, but they are suppressed by miR-203 mimics (Figures 4(i) and 4(j)). Accordingly, the Hoechst staining results have shown that obvious apoptosis occurred in SH-SY5 cells that have undergone H/R treatment versus control and that miR-203 mimics could significantly reduce the apoptosis of H/R-treated SH-SY5Y cells (Figures 4(k) and 4(l)). These results suggested that the insufficiency of miR-203 will lead to oxidative stress injury and its mechanism involving in the posttranscription regulation of ATF2.

### 3.5. Inhibition of ATF2 Ameliorates Oxidative Stress Injury via Regulating NOX2

**Figure 5:**
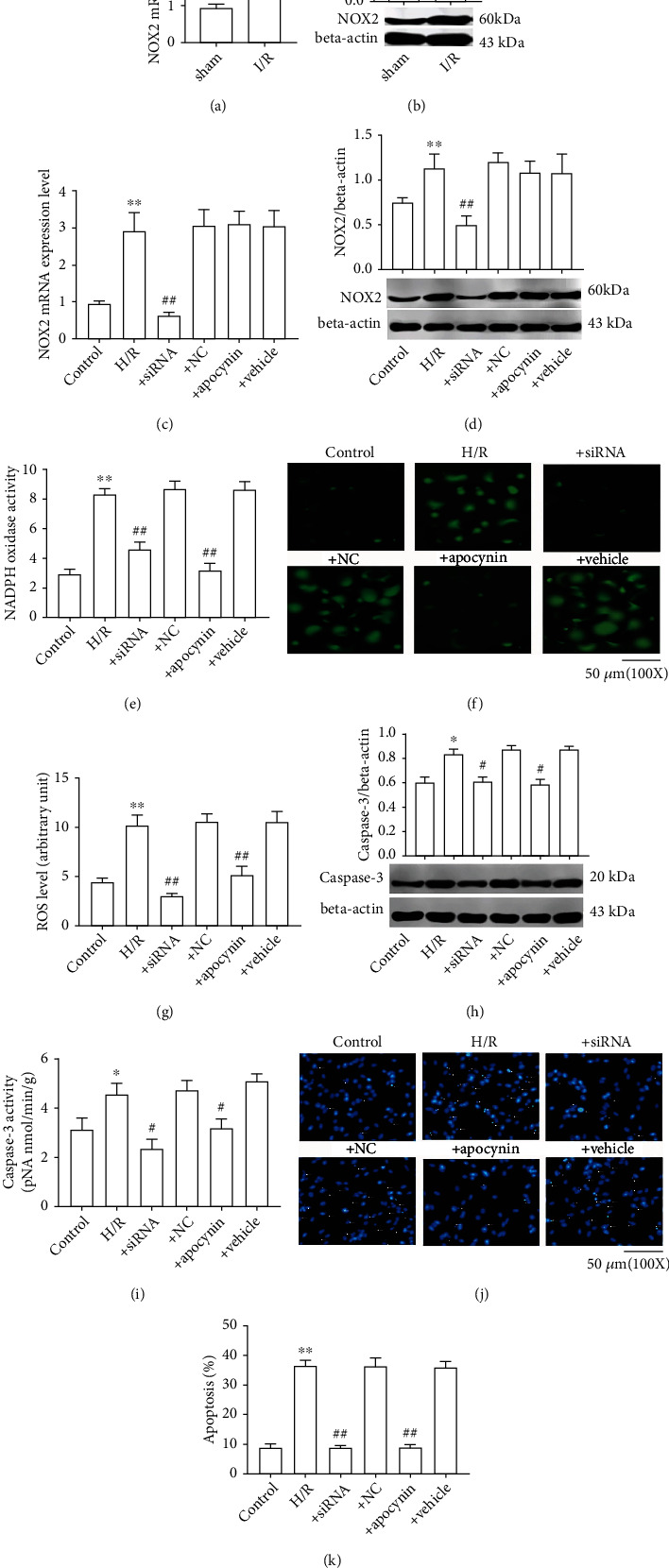
Inhibition of ATF2 ameliorates oxidative stress injury relating to NOX2 expression regulation. (a, c) mRNA level of NOX2; (b, d) protein expression of NOX2; (e) NOX enzyme activity; (f) representative images of ROS; (g) ROS level; (h) protein expression of caspase3; (i) caspase3 activity; (j) the detection of cell apoptosis by Hoechst staining; (k) percentage of cell apoptosis. ^∗^*P* < 0.05 vs. control or sham; ^∗^*P* < 0.05 vs. control or sham; ^∗∗^*P* < 0.01 vs. control or sham; ^#^*P* < 0.05 vs. H/R; ^##^*P* < 0.01 vs. H/R.

Considering that ATF2 is a transcription factor of NOX2, the effect of ATF2 on NOX2 expression was then evaluated. As the present data has shown, the expression levels of NOX2 in CI/R model rats or in H/R-induced cells were significantly upregulated, which were positively related to ATF2 (Figures 5(a)–5(d)). To confirm that ATF2 was involved in regulating the expression of NOX2, ATF2 was knocked down by using a siRNA against ATF2 and the siRNA was proved to work properly (figureS2). Expectedly, ATF2 knockdown obviously downregulated the NOX2 expression in H/R-induced cells (Figures 5(c) and 5(d)). This indicated that ATF2 has roles in NOX2 expression regulation. The results of NOX enzyme activity indicated that H/R induction significantly increased the total NOX activity, while knockdown of ATF2 significantly reduced its activity (Figure 5(e)). In accordance with the result of NOX activity, it is found that the ROS levels obviously increased in H/R-induced cells versus control and that ATF2 knockdown significantly inhibited the ROS level in H/R-induced cells (Figure 5(f)). As expected, apocynin intervention did not affect NOX2 expression in H/R-induced SH-SY5Y cells but significantly inhibited NOX enzyme activity and ROS levels (Figures 5(c)–5(f)). Next, the expression of caspase-3, as well as its activity in H/R-induced SH-SY5Y cells, was detected. Consistent with the ROS level, H/R induction obviously increased the expression level of caspase-3, as well as its enzyme activity in SH-SY5Y cells, while ATF2 knockdown significantly decreased these effects (Figures 5(g) and 5(h)). The apoptosis of cells was then examined via Hoechst staining. Consistently, H/R induction obviously aggravated the apoptosis of SH-SY5Y cells, while ATF2 knockdown obviously inhibited the apoptosis of these cells (Figures 5(i) and 5(j)). These data indicated that ATF2 aggravates oxidative stress injury by increasing the expression of NOX2.

### 3.6. Overexpression of lncRNA PINK1-AS Results in Oxidative Stress Injury by Regulating the miR-203/ATF2/NOX2 Axis in SH-SY5Y Cells

**Figure 6:**
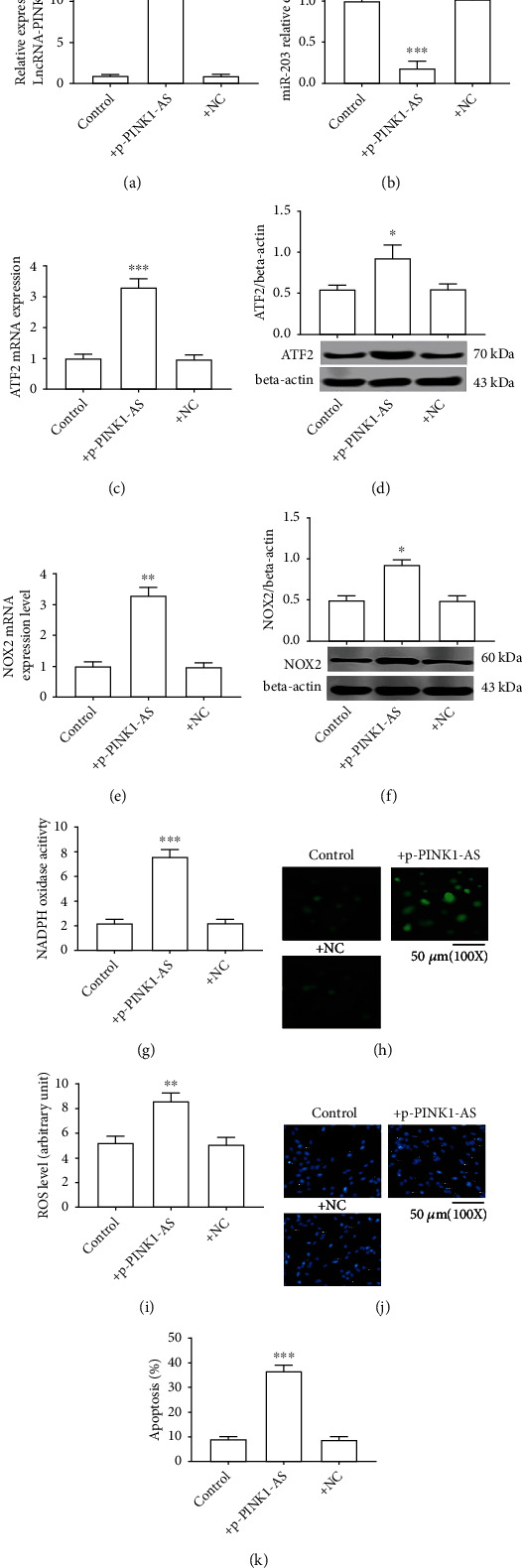
Overexpression of lncRNA PINK1-AS results in oxidative stress injury by regulating the miR-203/ATF2/NOX2 axis in SH-SY5Y cells. (a) lncRNA PINK1-AS expression level; (b) miR-203 expression level; (c) ATF2 mRNA expression; (d) ATF2 protein expression; (e) NOX2 mRNA expression; (f) NOX2 protein expression; (g) NOX enzyme activity; (h) presentative images of ROS level; (i) ROS level; (j) the detection of cell apoptosis by Hoechst staining; (k) percentage of cell apoptosis. ^∗^*P* < 0.05 vs. control or sham; ^∗∗^*P* < 0.01 vs. control; ^∗∗∗^*P* < 0.001 vs. control.

To further confirm that lncRNA PINK1-AS has roles in oxidative stress by regulating the miR-203/ATF2/NOX2 axis, a plasmid was constructed to overexpress lncRNA PINK1-AS (Fig S1) and its action in SH-SY5Y cells was observed. As the present data has shown, cells transfected with this lncRNA PINK1-AS plasmid showed significantly increased lncRNA PINK1-AS expression compared with the control group, while cells transfected with NC plasmid demonstrated no notable effect (Figure 6(a)). This indicated that the lncRNA PINK1-AS plasmid worked correctly. Next, related gene expression (miR-203, ATF2, and NOX2) was measured, and it was found that lncRNA PINK1-AS obviously decreased miR-203 expression but increased ATF2 and NOX2 expression in H/R-induced cells (Figures 6(b)–6(f)). Consistently, lncRNA PINK1-AS significantly increased the NOX activity in SH-SY5Y cells, as well as ROS level (Figures 6(g)–6(i)). Moreover, lncRNA PINK1-AS significantly increased the apoptosis level of cells (Figures 6(j) and 6(k)). These data further suggested that lncRNA PINK1-AS exerts important roles in oxidative stress injury in neurons via the regulation of the miR-203/ATF2/NOX2 axis.

## 4. Discussion

**Figure 7:**
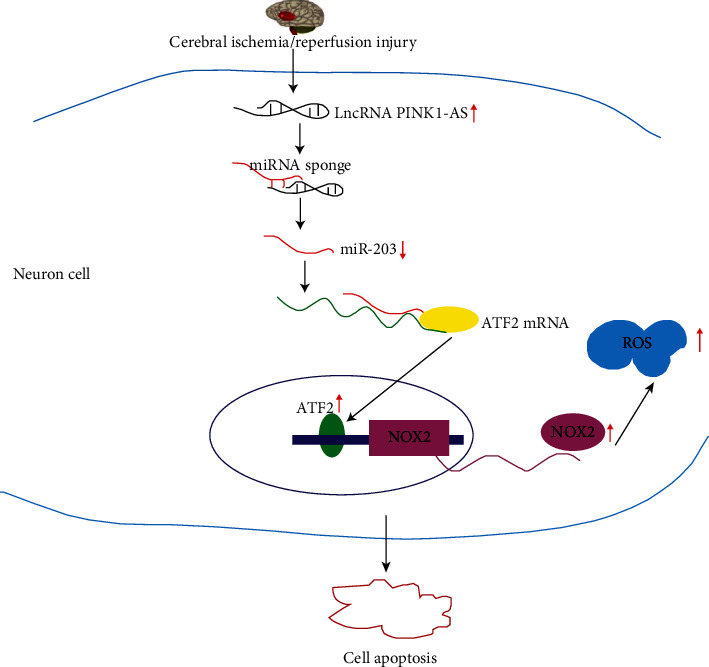
Sketch map of CI/R injury induced by lncRNA PINK1-AS. The expression of lncRNA PINK1-AS increased in brain tissues that have undergone CI/R injury. Highly expressed lncRNA PINK1-AS contributed to a low expression of miR-203 by acting as a sponge of miR-203. Low expression of miR-203 led to ATF2 expression upregulation. As a transcript factor of NOX2, highly expressed ATF2 contributed to high expression of NCF2 and NOX2, which led to ROS generation. Excessive ROS resulted in neuron cell apoptosis.

lncRNAs are a class of macromolecules with multiple biological functions and extensively participated in the pathophysiological process of ischemic stroke. The present results demonstrated that the lncRNA PINK1-AS, as a sponge for miR-203, aggravates oxidative stress injury that is due to cerebral ischemia followed by a short reperfusion, and its action mechanism involves highly expressed PINK1-AS endogenously competing with miR-203, thus, resulting in upregulation of ATF2 and NCF2 (both targets of miR-203), which leads to upregulation of NOX2 and generation of excessive ROS (Figure 7).

To observe the role of lncRNA in CI/R injury, a MCAO rat model that is subjected to a 2 h ischemia followed by a 24 reperfusion and a H/R SH-SY5Y cell model that is subjected to a 4 h hypoxia followed by a 20 h reoxygenation were established to simulate the occurrence of clinical ischemic stroke. Through microarray screening, it was found that PINK1-AS was obviously upregulated in CI/R injury tissues; this indicated that PINK1-AS may have roles in CI/R injury. PINK1-AS is a noncoding reverse translation chain of the PINK1 gene, which is widely expressed in neural tissues. Previous evidence has shown that PINK1-AS participated in the regulation of nerve cell mitochondrial function and was closely related to the pathophysiological process of numerous neurodegenerative diseases, including Parkinson's disease [10, 21]. In neurodegenerative diseases, PINK1 mutations or downregulation led to oxidative stress [22]. Some studies have shown that there was a specific association between PINK1-AS and PINK1 expression; for example, the downregulation of PINK1 expression will cause the upregulation of PINK1-AS expression [15]. This indicated that overexpression of PINK1-AS was related to CI/R injury. As a matter of fact, the current data proved that there was a positive correlation between highly expressed PINK1-AS level and excessively generated ROS level. Additionally, it was identified that knockdown of PINK1-AS significantly reduced ROS production in H/R-induced SH-Y5Y cells. This indicates that PINK1-AS has key roles in oxidative damage caused by CI/R. However, presently, the mechanism of PINK1-AS exacerbating oxidative stress injury is yet to be fully elucidated.

Recent evidence has shown that lncRNAs, as miRNA sponges, participated in the pathophysiological process of CI/R injury. Therefore, we hypothesized that PINK1-AS may promote oxidative stress by regulating miRNA expression. In fact, the present study firstly predicted the possible targets of PINK1 via bioinformatics analysis and found that there may be an interaction between miR-203 and PINK1-AS. This hypothesis has been confirmed by experiments that showed that miR-203 could obviously reduce relative luciferase activity. Previous investigations demonstrated that miR-203 is a multifunctional molecule, which is widely participating in the inflammatory response and oxidative stress of the heart and nervous tissue [14–17]. It is revealed that myocardial ischemia reperfusion injury can cause the downregulation of miR-203 expression in the heart tissue, and miR-203 overexpression or sevoflurane treatment can upregulate the expression of miR-203 and exert a protective effect on the myocardium, suggesting that miR-203 is a target for ischemic heart disease [15]. Li et al. [16] observed that the insufficiency of miR-203 was related to CI/R injury, and its specific mechanism involved the miR-203/Slug axis. Consistent with the findings of other researchers, the present data proved that miR-203 obviously decreased in IR-treated brain tissues. To observe whether miR-203 was involved in CI/R oxidative stress injury, the current study conducted a miR-203 function-gain experiment. As the data has shown, miR-203 mimics could obviously increase the expression level of miR-203 in H/R-induced neuron cells but decrease ROS levels, caspase-3 expression, and cell apoptosis. This suggests that miR-203 indeed participated in oxidative stress. Although emerging evidences have shown that miR-203 participates in the development of diabetic nephropathy by targeting regulating the expression of semaphorin 3A, resultingly inhibiting oxidative stress, or that it participates in the pathogenesis of diabetic cardiomyopathy by regulating the expression of PI3KCA, thereby inhibiting oxidative stress [23], as far as we know, no investigations have been performed on the molecular mechanism of miR-203 in CI/R oxidative stress injury. Hence, the current study predicted miR-203's targets and the bioinformatics prediction results have shown that ATF2 was a predicted target gene for miR-203 and this was confirmed experimentally. It was found that miR-203 mimics could obviously reduce the luciferase expression in cells that successfully expressed ATF2-WT plasmid. In line with the research results by Li et al. [24], this study proved that ATF2 is significantly upregulated in the CI/R group and that the ATF2 expression level negatively correlated with miR-203 expression level. In the miR-203 function-gain experiment, it was identified that miR-203 mimics obviously reduced the expression level of ATF2 in H/R-induced cells. This suggests that the miR-203/ATF2 axis has key roles in oxidative stress.

ATF2 has been demonstrated to participate in oxidative stress by numerous investigations. For instance, Liu et al. [25] have found that arsenic treatment upregulated the expression of ATF2 in urinary epithelial cells, and this process was mediated by ROS. Moreover, Banerjee et al. [26] observed that the antioxidant mechanism of resveratrol was involved in the regulation of p38 MAPK/ATF2/inducible nitric oxide synthase (iNOS) signaling. Considering that ATF2 belongs to the AP (activator protein) family and is a transcript factor of numerous genes (such as iNOS and glucose regulatory protein 78) and is regulated by several genes (such as JNK) [27], we hypothesized that ATF2 is highly likely to participate in NOX2 expression regulation. Bioinformatics analysis identified that ATF2 was a transcription factor of NOX2. This result is exciting, as our earlier studies found that the high expression of NOX2 was an important cause of CI/R oxidative stress injury [3, 28]. To confirm that ATF2 indeed promotes the expression of NOX2 under CI/R injury, ATF2 was knocked down to observe its effect. As expected, ATF2 knockdown significantly reduced NOX2 expression in H/R-induced SH-SY5Y cells. Different from the ATF2 knockdown results, apocynin treatment did not reduce the expression of NOX2 but inhibited NOX enzyme activity. These results suggested that reducing NOX2 expression and inhibiting NOX activity are two important methods to alleviate CI/R injury. However, ATF2 knockout did not completely block the expression of NOX2, suggesting that, in addition to ATF2 directly affecting the expression of NOX2, other factors participated in NOX2 expression regulation. For example, Zhang et al. [29] demonstrated that MLC kinase is involved in NOX2 expression regulation, while Hordijk [30] revealed that Rac was involved in NOX2 expression regulation. The current study identified that NFC2 also participated in NOX2 expression regulation. This indicates that the NOX2 expression regulation is a complicated biological process.

Although the relationship between long noncoding RNA PINK1-AS and oxidative stress was studied in animals and verified by cell experiments, it was not proved that knockdown of PINK1-AS could prevent CI/R injury. Considering that CI/R injury is the outcome of multiple factors, the results of in vitro experiments may not be applicable to in vivo animals. For further clarifying its biological function, knocking down PINK1-AS in vivo and observing its effect on cerebral ischemia-reperfusion injury are necessary. In addition, ATF2 is a transcription factor, and whether knocking down ATF2 will cause other effects besides oxidative stress remains to be further explored. These would be what we will do in our future studies.

In conclusion, as far as we know, the current study was the first to demonstrate that PINK1-AS accelerated CI/R-induced oxidative stress injury by acting as a sponge of miR-203. Moreover, it was found that the PINK1-AS/miR-203/ATF2 axis involving in NOX2 expression regulation, is a possible treatment target for CI/R oxidative stress injury.

## Figures and Tables

**Table 1:** Primers for real-time PCR.

Gene	Forward primer	Reverse primer
miR-203	5′-GGGGTGAAATGTTTAGGAC-3′	5′-CAGTGCGTGTCGTGGAGT-3′
U6	5′-CTCGCTTCGGCAGCACA-3′	5′-AACGCTTCACGAATTTGCGT-3′
lncPINK1-AS	5′-CCACGATACTTCACTGGGGC-3′	5′-GAACGTGGTTCCACTGTCCA-3′
ATF2	5′-CCCACCAGCTACAAAGTGTC-3′	5′-AGGGGCAGTGCATAGAAAGG-3′
NOX2	5′-ACAAGGTTTATGACGATGAGCC-3′	5′-TTGAGCAACACGCACTGGAA-3′
*β*-Actin	5′ CCCATCTATGAGGGTTACGC-3′	5′-TTTAATGTCACGCACGATTTC 3′

## Data Availability

The dataset used and/or analyzed during the current study is available from the corresponding author on reasonable request.

## Supplementary Materials

Supplementary Materials Figure S1: p-PINK1-AS recombinant plasmid construction. (A) Vector plasmid pCMV-Cre-EGFP; (B) schematic diagram of p-PINK1-AS recombinant plasmid construction; (C) plasmid electrophoresis. Figure S2: gene expression level. (A) lncRNA PINK1-AS expression level; (B) ATF2 mRNA expression; (C) miR-203 expression level. ^∗∗∗^*P* < 0.001 vs. control.
