# Supplementary material for: lncRNA PINK1-AS Aggravates Cerebral Ischemia/Reperfusion Oxidative Stress Injury through Regulating ATF2 by Sponging miR-203
Source: Oxid Med Cell Longev. 2022 Jul 9;2022:1296816. doi: 10.1155/2022/1296816 (PMC9288285; doi:10.1155/2022/1296816)

**FigureS1**

The figureS1 described the p-PINK1-AS recombinant plasmid construction and confirmation.


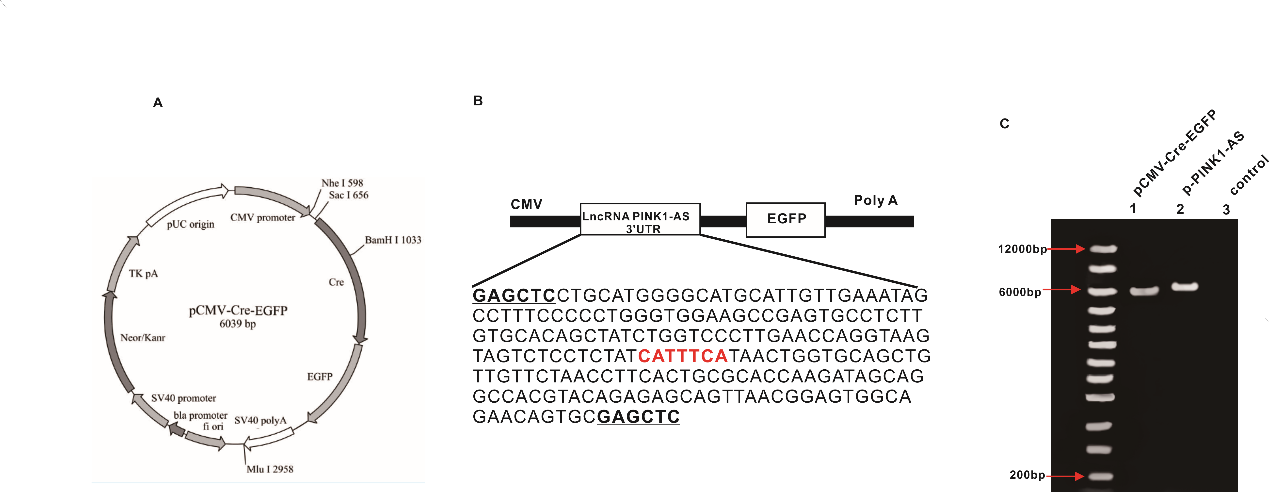


**FigureS2**

The figureS2 described the siRNA or mimic works properly.


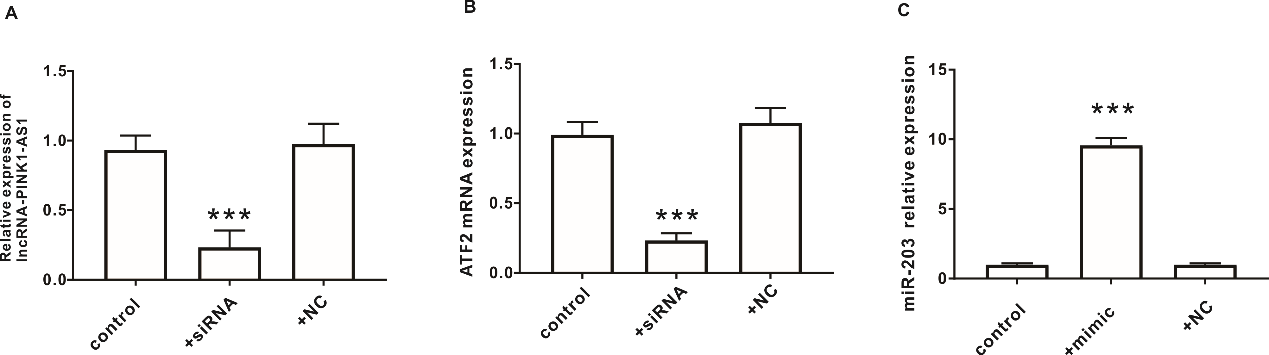

Supplement: Supplementary Materials — Figure S1: p-PINK1-AS recombinant plasmid construction. (A) Vector plasmid pCMV-Cre-EGFP; (B) schematic diagram of p-PINK1-AS recombinant plasmid construction; (C) plasmid electrophoresis. Figure S2: gene expression level. (A) lncRNA PINK1-AS expression level; (B) ATF2 mRNA expression; (C) miR-203 expression level. ∗∗∗P < 0.001 vs. control. [file 1296816.f1.docx]
